# Effects of *in ovo *electroporation on endogenous gene expression: genome-wide analysis

**DOI:** 10.1186/1749-8104-6-17

**Published:** 2011-04-28

**Authors:** Emma K Farley, Emily Gale, David Chambers, Meng Li

**Affiliations:** 1 MRC Clinical Sciences Centre, Hammersmith Hospital Campus, Imperial College London, W12 0NN, UK; 2 MRC Centre for Developmental Neurobiology, 4th Floor New Hunt's House, King's College, Guy's Campus, London, SE1 1UL, UK

## Abstract

**Background:**

*In ovo *electroporation is a widely used technique to study gene function in developmental biology. Despite the widespread acceptance of this technique, no genome-wide analysis of the effects of *in ovo *electroporation, principally the current applied across the tissue and exogenous vector DNA introduced, on endogenous gene expression has been undertaken. Here, the effects of electric current and expression of a *GFP-*containing construct, via electroporation into the midbrain of Hamburger-Hamilton stage 10 chicken embryos, are analysed by microarray.

**Results:**

Both current alone and in combination with exogenous DNA expression have a small but reproducible effect on endogenous gene expression, changing the expression of the genes represented on the array by less than 0.1% (current) and less than 0.5% (current + DNA), respectively. The subset of genes regulated by electric current and exogenous DNA span a disparate set of cellular functions. However, no genes involved in the regional identity were affected. In sharp contrast to this, electroporation of a known transcription factor, Dmrt5, caused a much greater change in gene expression.

**Conclusions:**

These findings represent the first systematic genome-wide analysis of the effects of *in ovo *electroporation on gene expression during embryonic development. The analysis reveals that this process has minimal impact on the genetic basis of cell fate specification. Thus, the study demonstrates the validity of the *in ovo *electroporation technique to study gene function and expression during development. Furthermore, the data presented here can be used as a resource to refine the set of transcriptional responders in future *in ovo *electroporation studies of specific gene function.

## Background

Determining the function of genes involved in embryonic development requires manipulation of gene expression in a spatially and temporally restricted manner. Whilst transgenesis in mice is used to this end, it is a time consuming and costly technique. Moreover, achieving the required spatiotemporal targeting via conditional transgenesis is not always achievable [1]. In contrast, the highly precise localisation and timing of expression construct insertion made possible by the *in ovo *electroporation technique provides an economical and efficient alternative to transgenesis [2]. These advantages make *in ovo *electroporation a widely used technique to study gene function during development.

*In ovo *electroporation is employed in both gain and loss of function studies and reporter studies to analyse gene function and regulation during development. This method has been applied to all areas of developmental biology, including neurogenesis and neural differentiation [3,4], axon outgrowth and guidance [5] axonal patterning in the developing limb [6], somitogenesis [7,8], skeletal muscle development [9] and eye development [10,11].

*In ovo *electroporation has been used to study development in mouse embryos [12] along with other vertebrates, including *Danio rerio *[13] and *Xenopus laevis *[13,14] and non-vertebrates such as *Drosophila melanogaster *[15] and Ascidiacea [16] (for reviews, see [17,18]). However, *in ovo *electroporation has been most widely applied to avian embryos because the avian embryo develops *in ovo *and has a planar topology. These characteristics greatly facilitate injection of the DNA construct, placement of electrodes and incubation of the electroporated embryo.

Independent of the experimental organism, electroporation involves microinjecting a gene expression construct, typically into a natural body cavity such as the neural tube. Subsequently, electrodes are placed flanking the site of injection and an electric current is applied across the embryo in the form of a rapid series of square wave pluses. This electric field transiently disrupts the stability of the plasma membrane, creating pores in the cell membrane. The negatively charged DNA constructs migrate towards the positive electrode and enter the cells in their path via these pores. The tissue adjacent to the negatively charged electrode remains untransfected, providing an internal control [19]. This method enables defined tissues to be targeted, by location of DNA injection and positioning of electrodes, at precise times of development. For more detailed information about specific parameters required refer to [2,20]. Further advances now enable focal electroporation and electroporation of different constructs in close proximity, increasing the precision and complexity of *in ovo *electroporation studies. This technique uses beads soaked in the DNA construct, which are microsurgically implanted into the embryo instead of microinjection [21].

*In ovo *electroporation is most commonly applied to Hamburger-Hamilton (HH) stage (st) 10 to 20 embryos. Embryos older than HH st20 have more compact tissues and increased tissue layers, making microinjection and *in ovo *electroporation more difficult. For older embryos *ex ovo *explant electroporation is an alternative method [22].

Typically, the construct used for *in ovo *electroporation contains the gene of choice along with *green fluorescent protein *(*GFP*), or another marker, in order to identify the cells that have taken up the construct. A popular construct to obtain transient expression is *pCAβ-IRES-GFP *containing the β-actin promoter, a cytomegalovirus (*CMV*) enhancer, a polylinker for inserting the desired gene followed by an internal ribosomal entry site (*IRES*) and *GFP *[23]. Expression of the translated product of the construct - for example, GFP - can be detected 2.5 hours after electroporation and peaks around 20 to 24 hours [24]. Expression of transient constructs can be maintained for 3 to 11 days [2,25]. Constitutive expression can be obtained by integrating plasmids into the genome using methods such as transposon-mediated gene transfer [26]. Using constructs with inducible promoters - for example, the tetracycline on tetracycline off system - enables further control over the timing of exogenous DNA expression [27]. It is also possible to use cell-type-specific enhancers [2].

In addition to overexpression studies, *in ovo *electroporation can also be used to carry out loss-of-function studies using RNA interference [28,29] or dominant negative constructs [30]. Constructs containing the gene of interest linked to either a repressor or an activator enable investigation of the transcriptional activity of genes *in vivo *[31]. As well as investigating gene function, *in ovo *electroporation can be used to study the activity of promoters during development *in vivo *using reporter constructs [32,33].

Traditionally, the downstream effects of *in ovo *electroporation of a gene of interest have been analysed using *in situ *hybridisation and antibody staining of a few select gene products. Parallel analysis of these genes in embryos electroporated with *GFP *alone is used as a control to identify any non-specific effects. This approach has the major disadvantage of surveying only a limited number of genes for qualitative transcriptional changes. To date, no effect of the *in ovo *electroporation technique on gene expression has been reported. However, evidence from other techniques, such as electrochemotherapy and electrogene therapy on malignant melanoma cells, indicates that both electric current and exogenous expression of DNA have a small but significant affect on endogenous gene expression [34].

The other potentially influential component of *in ovo *electroporation is the introduction of DNA constructs, which results in significant levels of exogenous DNA within the electroporated cells. The cellular response to expression of plasmid DNA has been analysed in several cell lines: Chinese hamster ovary epithelial cells (CHO-K1), mouse fibroblast cells (NIH3T3), human embryonic kidney cells (HEK293) and several melanoma lines [35]. Exogenous DNA expression in these cell lines was shown to have differing affects, triggering a DNA damage response in CHO-K1 and NIH3T3 cells but causing no cellular response in HEK293 cells or melanoma cell lines [35]. These differences in response to exogenous DNA expression are postulated to be due to species differences [35]. How chicken cells respond to exogenous DNA expression or indeed how multicellular living organisms respond is yet to be examined.

Despite these reports suggesting exposure to current and exogenous DNA within cells can cause changes in endogenous gene expression, there has been no systematic analysis of the effects of either of these components within the developing embryo. Understanding the experimentally induced changes caused by *in ovo *electroporation has recently become extremely pertinent since the sequencing of the chicken genome and the availability of a highly representative chicken genome microarray (Affymetrix GeneChip Chicken Genome Array) now enables the coupling of *in ovo *electroporation to genome-wide analysis. This strategy provides an opportunity to use *in ovo *electroporation to unravel the role of genes during the embryonic development in a high-throughput and genome-wide manner. If this strategy is to be used, a better understanding of the effects of this technique are required. To address this, we carried out microarray analysis of the effects of both the electric current and exogenous DNA expression associated with *in ovo *electroporation.

## Results

### Experimental strategy

**Figure 1:**
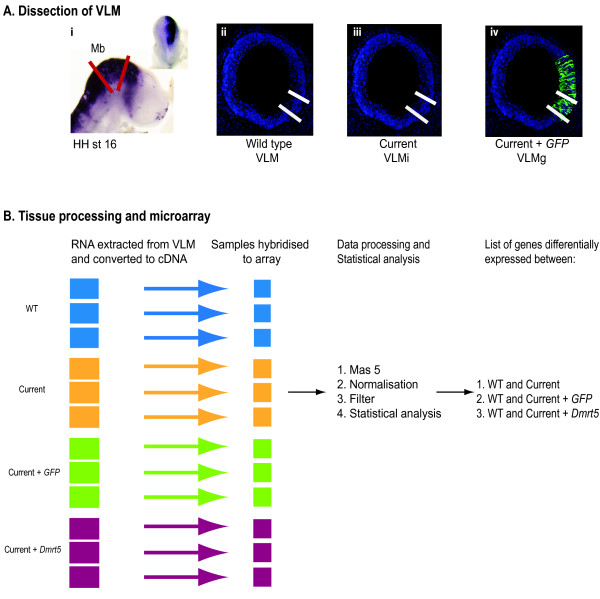
**Experimental strategy**. **(A) **Dissection of the ventral lateral midbrain (VLM) region. (i) *In situ *sagital view of a HH st16 electroporated embryo expressing *pCAβ-IRES-Dmrt5 *construct (dorsal view in the inset). Red lines indicate the midbrain (Mb) region, which was dissected out. (ii-iv) Coronal sections of midbrain. White lines mark the VLM region. This region was isolated from (ii) control embryos (VLM), (iii) VLM exposed to current (VLMi), (iv) VLM exposed to current + *GFP *(VLMg), and VLM exposed to current + *Dmrt5 *(VLMd; image not shown) for investigation of transcriptional profiles by microarray analysis. (**B) **Tissue processing and microarray analysis. Six VLM tissues were pooled for each biological replicate, and three biological replicates were used for each condition. cDNA was isolated from these pools and hybridised to the Affymetrix Chicken Genome Array. Following MAS5, normalisation and filtering, genes whose expression differed significantly between the wild type (WT; VLM) and VLMi, VLMg and VLMd were identified by one-way ANOVA.

To investigate the effects of the electric current and exogenous DNA expression associated with *in ovo *electroporation on endogenous gene expression of the developing chicken embryo, we used microarray analysis to compare the gene expression profile of four pooled samples of ventral lateral midbrain (VLM) tissue. The four samples were unelectroporated VLM (VLM), VLM exposed to electric current only (VLMi), VLM exposed to expression of *GFP *and electric current (VLMg) and VLM exposed to exogenous expression of a regulatory gene, *Dmrt5 *(*Doublesex and Mab related transcription factor like 5*) and electric current (VLMd) (Figure 1). The current was generated with an ECM830 Electro Square Porator (5 × 50-ms pulses of 12 V). The GFP construct used was *pCAβ-IRES-GFP*. Electroporation was carried out at HH st10, and embryos were collected 24 hours later. Thus, our experiment was specifically designed to interrogate the set of genes 'stably' regulated following electroporation, rather than those responding to the initial treatment. We reasoned that genes identified at this 24 hour time point would be more likely to have a significant contribution to altering long-term cell identity as opposed to those genes whose expression changed rapidly in the minutes following electrical insult.

### Effect of *in ovo *electroporation on endogenous gene expression

**Figure 2:**
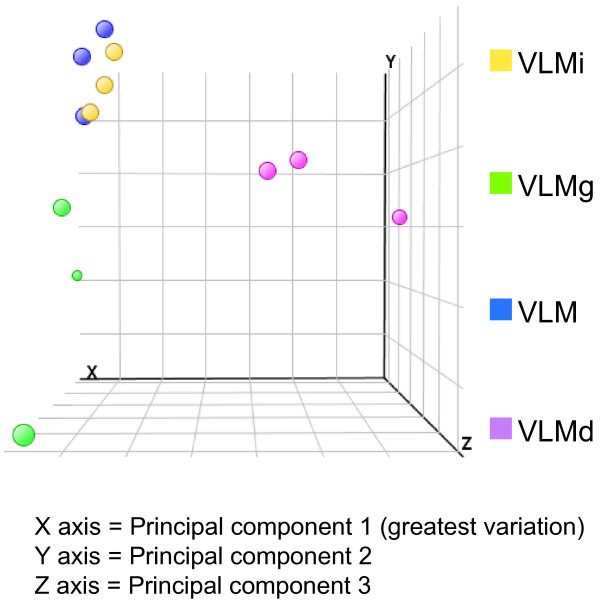
**Principal component analysis to identify genome-wide transcriptional variation caused by *in ovo *electroporation**. This plot shows the variation between the samples; each dot represents the global gene expression of a single microarray. The greatest variation is measured on the x-axis, then the y- and z-axes, respectively. Wild-type VLM, VLMi and VLMg samples cluster together on the x-axis, indicating that there is little variation in the gene expression between these three conditions. VLMd, in which the regulatory gene *Dmrt5 *is exogenously expressed, clusters separately, indicating larger genome-wide variation between this sample and the others.

The distribution of gene expression values across the whole microarray was monitored before and after normalisation by quantile grouping (box plots) and was found to be similar for each set of biological replicates (data not shown). The variation in biological replicates was also analysed by principal component analysis (PCA), which measures the variation in expression levels between the microarrays. PCA showed all biological replicates cluster together, demonstrating that the biological replicate datasets are similar and reproducible. As well as biological replicates clustering together, three experimental conditions, VLM, VLMi and VLMg, cluster together, indicating there is little difference in gene expression between these three conditions. In contrast, exogenous expression of the regulatory gene *Dmrt5 *caused the VLMd samples to cluster separately from the other conditions (Figure 2). This preliminary analysis of global gene expression levels indicates that exposure to current or current + *GFP *has little effect on endogenous gene expression, whilst addition of a regulatory gene causes a much larger change in endogenous gene expression.

Analysis of the array data to identify genes that show significant differential expression (*P *> 0.05, >2.0-fold change) upon exposure to current, current + *GFP *or current + *Dmrt5*, when compared to the unelectroporated tissue, identified that only 41 of the 32,773 oligonucleotides spotted on the array were changed by exposure to current and 176 by current + *GFP*. This equates to a change in gene expression of 0.1% (current) and 0.5% (current + *GFP)*, of the oligonucelotides found on the array, respectively. By comparison, exogenous expression of a regulatory gene, *Dmrt5*, led to a significant change (*P *> 0.05, >2.0-fold change) in the expression of 479 oligonucleotides or 1.5% of the oligonucleotides on the array. For further analysis, these oligonucleotide lists were processed to remove probe set duplicates and oligonucelotides that did not correspond to annotated genes (Table 1).

**Table 1:** Differential expression of genes upon exposure to current, current + *GFP *and current + *Dmrt5*

Condition	Number of oligonucleotides differentially expressed	Percentage of oligonucleotides showing differential expression	Number of annotated endogenous genes differentially expressed
Current	41	0.13	21
Current + *GFP*	176	0.54	111
Current + *Dmrt5*	479	1.46	309

### Effect of current on endogenous gene expression

**Figure 3:**
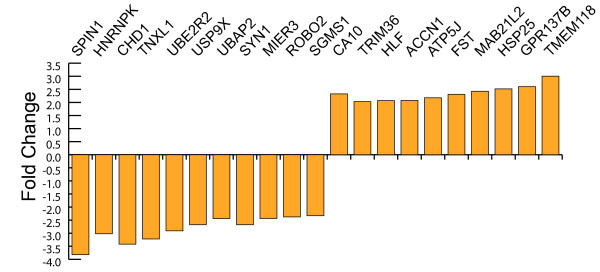
**Genes showing differential expression in response to current**. Genes showing differential expression when exposed to current and their fold change.

Only 41 of the 32,773 oligonucelotides were significantly differentially expressed 24 hours after exposure to current alone. This corresponds to 21 annotated endogenous genes (Figure 3). There was an equal proportion of up- and down-regulated genes (10 and 11, respectively) and the range of their fold changes was -3.8 to 3.0 (Table 2). Thus, the application of an electric current alone to developing neuronal cells within the ventral midbrain has a small effect on the constituents of the cell transcriptome. These findings are consistent with other *in vitro *studies that suggest current alone has a minimal impact on cellular identity and specification [34].

**Table 2:** Genes showing differential expression upon exposure to current and their fold change

Gene name	Fold change
*SPIN1*	-3.8
*HNRNPK*	-3.0
*CHD1*	-3.4
*TXNL1*	-3.2
*UBE2R2*	-2.9
*USP9X*	-2.6
*UBAP2*	-2.4
*SYN1*	-2.6
*MIER3*	-2.4
*ROBO2*	-2.3
*SGMS1*	-2.3
*CA10*	2.3
*TRIM36*	2.0
*HLF*	2.0
*ACCN1*	2.0
*ATPJ5*	2.1
*FST*	2.3
*MA21L2*	2.4
*HSP25*	2.5
*GPR137B*	2.6
*TMEM118*	3.0

We next looked at the composition of the 21 responders in this group to determine the set of cellular activities affected. The 21 responders belong to a disparate array of biological functions (Table 3). The small number of genes meant it was not possible to identify biological processes that were significantly enriched in this population using Gene Ontology (GO) analysis.

**Table 3:** Biological functions of genes that responded to current

Biological category	Genes
Response to oxidative stress	*Hnrnpk*, *Usp9x*, *Hlf*, *Txn1l*, *Hsp25*
Response to pH/redox changes	*Ca10*, *Atp5j*, *Accn1*, *Txn1l*
Regulation of apoptosis	*Accn1*, *Hlf*, *Usp9x*, *Hnrnpk*, *Sgms1*

Upregulation of *Heat shock protein 25 *(*Hsp25*) was observed. Two members of the ubiquitination pathway, *uber2r *and *ubap2*, also responded upon exposure to current. Five of the 21 responders are involved in response to oxidative stress. However, no major oxidative stress components show a change in expression. Four of the 21 genes are reported to respond to pH/redox changes. Five responders are involved in regulation of apoptosis, either to promote (two genes) or inhibit (three genes) this process. For all three of these biological functions, regulators of processes rather than determiners are affected, suggesting that the effect of the electric current on biological processes is minimal.

### Effect of current and vector DNA on endogenous gene expression

The set of genes derived from the control tissues (VLM) was compared with their electroporated counterparts expressing *GFP *(VLMg). This analysis revealed that only 176 of the 32,773 oligonucleotides represented were significantly differentially expressed (*P *> 0.05, >2.0-fold change) which corresponds to less than 0.5% of the transcriptome. This corresponds to 111 endogenous genes (Table 4). Of these 111 responders to current + *GFP*, only 10 were upregulated, with the remaining 101 being downregulated. All of the upregulated genes showed a 2.0- to 3.3-fold change except for *BSFB *(*beaded filament structural protein1*, *filensin*), which was upregulated 7.8-fold. BSFB is a filament protein and forms a structural constituent of eye lens [36], and the significance of its upregulation is not known. All downregulated genes showed between a 2.0- and 3.6-fold change in expression. Thus, the application of an electric current in combination with expression of exogenous DNA in the form of *GFP *has a greater effect on neuronal cells than current alone. However, this is still a much smaller effect than exogenous expression of a regulatory gene, such as *Dmrt5*, which causes a change in gene expression of 309 endogenous genes.

**Table 4:** Genes showing differential expression upon exposure to current + *GFP *and their fold change

Gene name	Fold change
*MYH10*	-2.0
*RCBTB1*	-2.0
*BAZ1B*	-2.0
*ZFHX3*	-2.0
*EP400*	-2.0
*GSPT1*	-2.0
*YLPM1*	-2.0
*ANKHD1-EIF4EBP3*	-2.0
*SGMS1*	-2.0
*MLL*	-2.1
*WAC*	-2.1
*PITPNM3*	-2.1
*TNRC6B*	-2.1
*SMPD3*	-2.1
*EPN2*	-2.1
*CCDC88A*	-2.1
*LOC427360*	-2.1
*TLN2*	-2.1
*DGCR8*	-2.1
*LOC418437*	-2.1
*TMTC3*	-2.1
*NIPBL*	-2.1
*PTCHD1*	-2.1
*BEND7*	-2.1
*GNPTAB*	-2.1
*SENP5*	-2.1
*NKTR*	-2.1
*FBXW8*	-2.1
*NISCH*	-2.1
*ACPL2*	-2.1
*LOC429468*	-2.2
*KCNK5*	-2.2
*TBL1X*	-2.2
*UBE4B*	-2.2
*NTRK3*	-2.2
*MLL3*	-2.2
*ALS2*	-2.2
*PHIP*	-2.2
*PPP6R3*	-2.2
*SPEN*	-2.2
*ERC1*	-2.2
*TJP1*	-2.2
*ABCB11*	-2.2
*PRPF38B*	-2.2
*LRRN1*	-2.2
*IL17RD*	-2.2
*TXNL1*	-2.3
*CHD1*	-2.2
*USP40*	-2.3
*PLXNB2*	-2.3
*18s rRNA*	-2.3
*SPTBN1*	-2.3
*PCDH19*	-2.3
*PDCD6*	-2.3
*FZD7*	-2.3
*RASGEF1A*	-2.3
*NAV2*	-2.3
*EP300*	-2.3
*GLS*	-2.4
*ATRX*	-2.4
*FAM130A2*	-2.4
*MDN1*	-2.4
*RPRD1A*	-2.4
*VCAN*	-2.4
*ARHGAP18*	-2.4
*JAG1*	-2.4
*BAZ2B*	-2.4
*HNRNPK*	-2.5
*CKAP5*	-2.5
*SYNE2*	-2.5
*ASPM*	-2.5
*PLA2G2E*	-2.6
*FIGN*	-2.6
*HERC2*	-2.6
*TMEM121*	-2.6
*SALL3*	-2.6
*TRAF3*	-2.6
*AKAP13*	-2.6
*RBM25*	-2.7
*LOC776927*	-2.7
*SON*	-2.7
*ARHGAP17*	-2.7
*AKAP9*	-2.8
*VGLL4*	-2.8
*ZEB2*	-2.8
*LRP2*	-2.8
*Ube2r2*	-2.9
*UBAP2*	-2.9
*YTHDC1*	-3.1
*TRIO*	-3.1
*GUCY1A2*	-3.3
*LMO7*	-3.3
*PDZRN3*	-3.4
*SFRS18*	-3.4
*HMGA2*	-3.5
*PRTG*	-3.5
*FHOD3*	-3.5
*VCL*	-3.6
*SPIN*	-3.6
*DLG1*	-3.7
*CACNG3*	2.0
*DNTTIP1*	2.0
*HUS1*	2.0
*PSPH*	2.5
*KCTDC7*	2.8
*WNT3A*	2.8
*EIF4A1*	3.1
*EGFL7*	2.9
*CIRBP*	3.3
*BFSP1*	7.8

**Figure 4:**
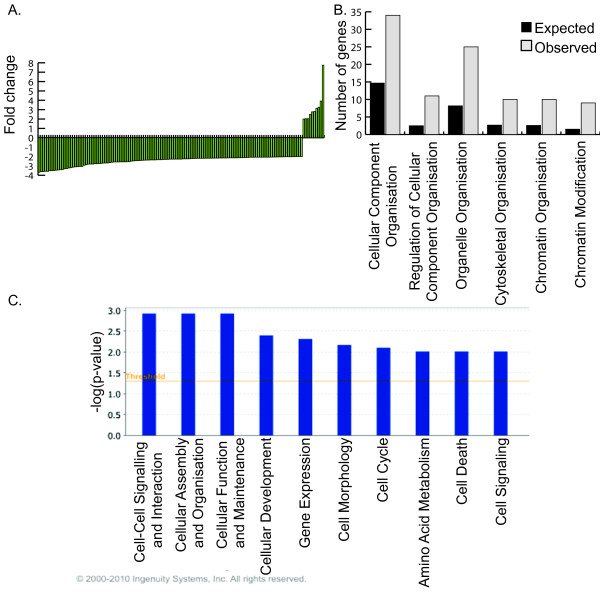
**Effect of electric current + *GFP *expression on endogenous gene expression of the VLM**. **(A) **Genes showing differential expression when exposed to current + *GFP *and their fold changes. For gene names refer to Table 3. **(B) **GOTM analysis showing biological function and expected and observed number of genes in each category compared to the number expected from a random set of 111 genes. **(C) **IPA functional analysis showing the top ten biological functions enriched in the VLMg compared to VLM samples.

We next looked at the composition of the 111 responders in this group to determine the cellular activities affected. GO Tree Machine (GOTM) analysis was used to identify biological processes that were significantly enriched in this population. The biological function 'cellular component organisation' was significantly affected by exposure to current + *GFP*. Within this category 'regulation of cellular component organisation', 'organelle organisation' and 'cytoskeletal organisation' were significantly affected. These changes in 'organelle organisation' included the subterms 'chromatin organisation' and 'chromatin modification', which were significantly over-represented in the tissue exposed to current + *GFP*. This indicates that current + *GFP *may affect the cellular organisation (Figure 4A). This finding was also identified by Ingenuity Pathway Analysis (IPA). Using IPA, the most significant biological functions within the list of responders are 'cell-cell signalling and interaction', 'cellular assembly and organisation' and 'cellular function and maintenance' (Figure 4B). 'Cell death' is the ninth most significant pathway, with 9 out of the 111 genes found to be involved in this process. However, the changes in these apoptotic genes include ones that could both promote and reduce apoptosis (Figure 4B).

**Figure 5:**
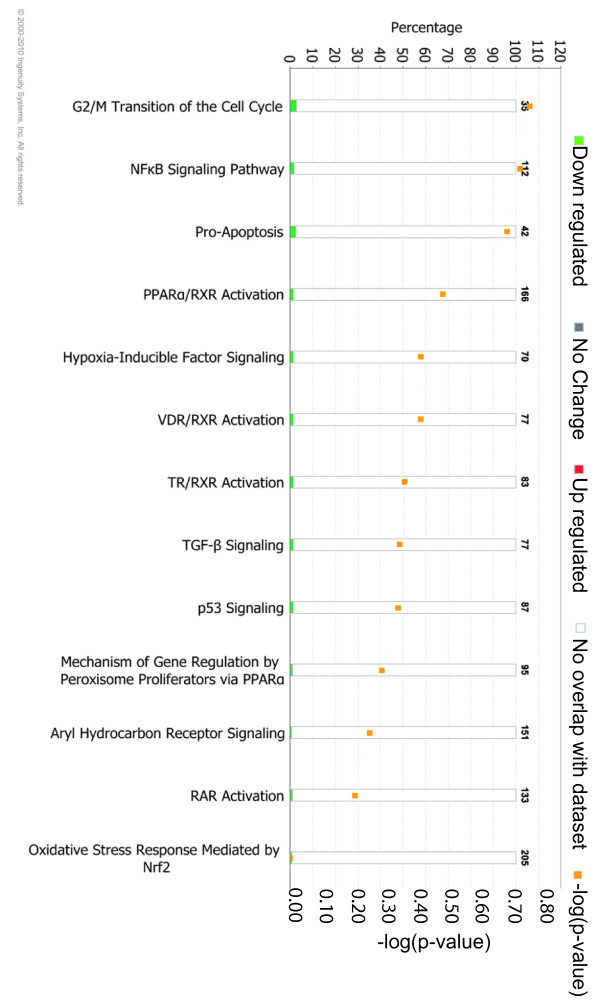
**Ingenuity Pathway Analysis toxicity analysis**. The genes showing differential expression upon exposure to current + *GFP *are not significantly involved in any type of toxicity. The bar chart shows the percentage of genes involved in each type of toxicity (left axis). Numbers above the bars are the number of genes that would equate to 100% for each type of toxicity. The orange points show the -log(*P*-value) (right axis) for each category, and thus indicate whether a significant number of genes are found within a category to suggest an actual toxicity affect. A -log(*P*-value) of 1.3 would be considered significant; all -log(*P*-values) are lower than 1.3 and therefore not significant.

To identify if any of the changes in gene expression upon exposure to current + *GFP *elicit a toxic affect, such as oxidative stress or apoptosis, IPA toxicity analysis was used (Figure 5). This analysis found some genes involved in pro-apoptosis and p53 signalling in the set of 111 responders. However, the number found is less than would be expected to occur in a random set of 111 genes, suggesting this is not statistically significant. Thus, *in ovo *electroporation does not induce toxic effects within the embryo as defined by the pathway analysis software.

### *In ovo *electroporation does not affect the regional identity of the VLM

**Figure 6:**
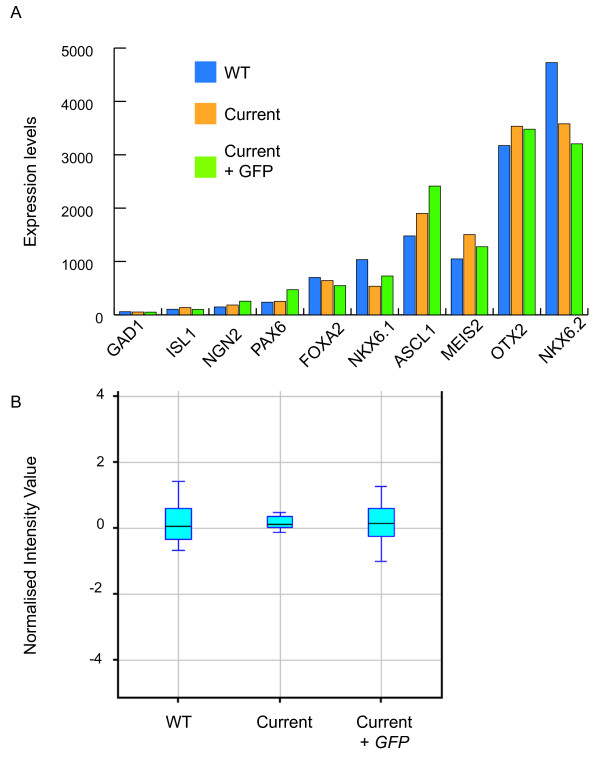
**Regional identity of VLM unaffected by current alone or current + *GFP***. **(A) **Expression profile of ten marker genes of the VLM obtained from the microarray analysis of VLM (wild type (WT)), VLMi and VLMg. In all conditions, marker genes are seen at the expected levels, and there is little difference between the three conditions. **(B) **Box plot showing the normalised expression values for all ten marker genes. All conditions have the same median and error bars overlap, indicating that the ten marker genes are not significantly differentially expressed between the three conditions. This shows the regional identity is not significantly affected by exposure to current or current + *GFP*. Statistical analysis using one-way ANOVA also shows that these marker genes are not differentially expressed.

Given the high number of patterning marker genes that are well described for the VLM, we were able to analyse the effects of current and expression of *GFP *on patterning of this region. The VLM is lateral to the floor plate, in the Nkx6.2 expressing region; therefore, we expected markers such as *Nkx6.2 *to be present in the microarray. Analysis of the transcriptional profile obtained for the VLM was reflective of the known genetic profile of the region and the expression levels of the ten marker genes were similar in the VLM, VLMi and VLMg samples (Figure 6A).

Indeed, statistical analysis using one-way analysis of variance (ANOVA) shows that these marker genes are not differentially expressed between the three samples to a *P*-value of 0.05. These data are illustrated in Figure 6B, which is a box plot of all ten marker genes. All the box plots have almost the same mean value and all overlap (Figure 6B). These data demonstrate that neither electric current nor current + *GFP* affect the regional identity of the tissue.

## Discussion

*In ovo *electroporation is an extremely powerful technique to investigate the function of genes and regulatory regions during development. With advances in methods used to analyse electroporated embryos, such as microarray analysis and next generation sequencing, a better understanding of the effects of this technique are necessary. To this end we carried out a genome-wide analysis of the effect of this technique on endogenous gene expression.

Our analysis has established that the electric current used during electroporation (5 × 50-ms pulses of 12 V) has a minimal affect on gene expression, causing a change in expression of only 21 endogenous genes. The upregulation of *Hsp25*, which encodes a heat shock protein known to be upregulated 24 hours after heat shock in response to protein aggregation [37], combined with changes in two members of the ubiquitination pathway indicate that some protein denaturation may have occurred upon exposure to current.

We have also established that the current and expression of exogenous DNA, in the form of *GFP*, has a small but statistically significant effect on gene expression, with no toxicity pathways significantly activated. The expression of exogenous DNA has a greater affect on endogenous gene expression than exposure to current alone, with 111 genes affected by exposure to current + *GFP*.

Interestingly, exogenous DNA expression leads to the downregulation of 101 genes, in contrast to the upregulation of 10 genes, suggesting that exogenous DNA expression, in these conditions, results in a repression of endogenous gene expression.

There has been surprisingly little investigation into the cellular response to exogenous DNA expression. *Hus1*, a gene involved in the ataxia telangiectasia-mutated (ATM) and ATM and Rad3-related signalling network, has been shown to be upregulated in response to exogenous DNA expression in CHO-K1 and NI3T3 cells [35] and in response to current + *GFP *in this experiment. IPA and GOTM analysis indicate that the exogenous expression of DNA causes changes in 'cellular organisation' functions, including 'cytoskeletal organisation' and 'chromatin organisation'. Despite the downregulation of several genes involved in 'chromatin organisation and modification', this does not appear to have had an appreciable effect on gene expression since only 0.5% of the genes show a change in gene expression after exposure to current + *GFP*.

The absence of any effect on the regional identity of the VLM indicates that any changes seen were not sufficient to affect the specification of the tissue at the time point investigated here.

In contrast to the above findings, current + *Dmrt5 *has a far larger affect on gene expression, causing a change in expression of 309 genes. These changes in gene expression are associated with cell specification, a known function of Dmrt5 (unpublished data). Comparison of the range of fold changes seen in genes affected by current + *Dmrt5 *(-15 to +1,125), current (-3.8 to +3.0) or current + *GFP *(-3.7 to +7.8) further highlight the minimal affect that the current and exogenous DNA expression used in this technique have on endogenous gene expression when compared to the affect seen when a regulatory gene is exogenously expressed.

**Figure 7:**
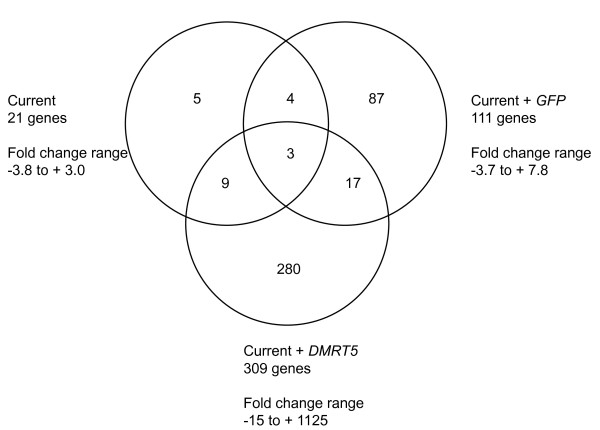
**Venn diagram showing overlap between genes differentially expressed in all three conditions**. Venn diagram showing annotated endogenous genes that are differentially expressed in all three conditions - current (VLMi), current + *GFP *(VLMg) and current + *Dmrt5 *(VLMd) - and the fold change range for each condition.

Three genes are affected in all experimental conditions, current, current + *GFP *and current + *Dmrt5 *(Figure 7). These are *Hnrnpk *(*Heterogeneous nuclear ribonucleoprotein K*), *Chd1 *(*Chromodomain-helicase-DNA-binding protein 1*) and *Txnl1 *(*Thioredoxin-like protein 1*). Hnrnpk is an RNA binding protein known to associate with pre-mRNAs in the nucleus and influences RNA processing, metabolism and transport. It is involved in maintaining cellular ATP levels in stress conditions, possibly by protecting its target mRNAs [38]. Chd1 is a chromatin remodelling enzyme that regulates transcription [39], and Txnl1 is a redox sensor [40].

Of the 21 genes affected by current, 7 are also affected by current + *GFP *and 12 are also affected by current + *Dmrt5 *(Figure 7). This indicates that the changes identified in response to current are reproducible, but that the addition of DNA does alter the tissue's response to the current. Of the 111 genes affected by current + *GFP*, only 19 are affected by exogenous expression of *Dmrt5 *(Figure 7). This again indicates that the genes responding to the experimental conditions are affected in a combinatorial nature.

## Conclusions

We have investigated the effects of both the current required for *in ovo *electroporation and the effect of exogenous DNA expression resulting from *in ovo *electroporation. The results of microarray analysis show that current and current + *GFP *have a small effect on endogenous gene expression, without affecting regional identity. This study demonstrates the validity of the *in ovo *electroporation technique to study gene function and expression within the developing embryo.

## Materials and methods

### *In ovo *electroporation

HH st10 embryos were windowed and electrodes place either side of the developing head (CUY610P1.5-1, Sonidel Ltd (Dublin, Ireland). A solution containing 7 μg/μl DNA construct *pCAβ-IRES-GFP or pCAβ-IRES-Dmrt5*, 2% polyvinyl alcohol, 0.05% Fast Green in water was injected into the developing midbrain. Five 12 V square wave pulses of 50 ms duration with an interval of 100 ms were applied across the electrodes using an ECM830 Electro-S Square Porator (BTX Inc. (Hawthorne, NY, USA). Following exposure to current, embryos were incubated at 37°C for 24 hours; embryos were collected at HH st16.

### Tissue preparation

Collected embryos were washed in PBS and transferred to clean PBS for dissection of the midbrain region, as marked by red lines in Figure 1A. Dissected tissue was transferred to 2 U/ml dispase (Sigma (St Louis, MO, USA) for 10 to 15 minutes. Mechanical dissection was used to remove the neural tube from the mesoderm. VLM was dissected from wild-type embryos (VLM), embryos that had experienced electric current (VLMi), embryos exposed to current and *GFP *(VLMg) and embryos exposed to current and the regulatory gene *Dmrt5 *(VLMd); Figure 1A(ii-iv) shows schematics of the region isolated. Only embryos showing high expression of GFP were used; Figure 1A(i) shows an *in situ *of an electroporated embryo, and Figure 1A(ii-iv) show sections of electroporated embryos, highlighting the electroporation efficiency and region that was dissected from the embryos.

For each microarray, six VLM, six VLMi, six VLMg or six VLMd tissue samples were collected and pooled. Total RNA was isolated from each pool (Absolutely RNA Miniprep Kit, Stratagene (La Jolla, CA, USA). RNA was sent to UK Bioinformatics for processing and analysis. (London, UK) The procedure used was as follows. cDNA was biotin labelled and samples were hybridized to Affymetrix GeneChip Chicken Genome Array according to the manufacturer's protocol. For each condition three sets of pooled samples were collected and hybridised to three arrays to obtain three biological replicates for each condition.

### Bioinformatics

Probe levels were calculated from raw data using the MAS5 algorithm embedded into the GCOS suite (version 1.2; Affymetrix). Data were analysed using the GeneSpring package (version GX7; Agilent Technologies, Wokingham, Berkshire, UK). The suitability of the expression data sets for inclusion in the analysis and the overall relationship between biological replicates was assessed using quantile plots and PCA. Samples were first normalised to the 50th percentile (median) across the entire expression data set. Genes were then filtered to remove any genes absent in all arrays. To identify genes showing differential expression between the three samples, a one-way ANOVA with no multiple testing correction was carried out. A *P*-value of 0.05 and a two-fold change in gene expression was used to determine genes showing differential expression between the samples.

### Ingenuity network analysis

The differentially expressed genes were analysed using IPA v8.0-2602 (Ingenuity Systems, Redwood City, CA, USA). The Ingenuity Knowledge Base is the largest knowledge base of its kind, with millions of findings curated from the full text literature. For analysis, the Affymetrix Chicken Genome Array gene list was used as a reference set. All data sources, all species, and all tissues and cell lines were used for the analysis. IPA uses a Fisher's exact test to determine which toxicity pathways and biological functions were significantly enriched within the set of genes showing differential expression compared to the entire list of genes represented on the array.

### Gene Ontology Tree Machine

GO provides a controlled vocabulary of about 20,000 terms in three independent hierarchies for cellular components, molecular functions and biological processes. The genes showing differential expression upon exposure to current or current + *GFP *were converted into mouse annotation and input into the GOTM program. As a reference set the entire mouse genome was chosen. The GOTM web-based tool carries out statistical analysis to identify enriched GO categories for the input gene sets and generates a GO tree to visualize GO terms that are enriched in the input gene list. Hypergeometric test was used to select enriched GO terms for each cluster compared to the GO terms of the entire mouse genome. A GO category was considered as enriched if the *P*-value was <0.01.

### *In situ *hybridisation

Whole mounts were exposed to digoxigenin-tagged antisense RNA mouse *Dmrt5 *probe overnight at 70°C for hybridisation with the exogenous *Dmrt5 *following the protocol described in [41].

## Abbreviations

CHO-K1: Chinese hamster ovary epithelial cells; Dmrt5: Double-sex and mab3-related transcription factor like 5; GFP: green fluorescent protein; GO: Gene Ontology; GOTM: Gene Ontology Tree Machine; HEK293: human embryonic kidney cells; HH: Hamburger-Hamilton; IPA: Ingenuity Pathway Analysis; IRES: internal ribosomal entry site; NIH3T3: mouse fibroblast cells; PBS: phosphate-buffered saline; PCA: principal component analysis; st: stage; VLM: ventral lateral midbrain; VLMd: ventral lateral midbrain exposed to current and *Dmrt5*; VLMg: ventral lateral midbrain exposed to current and *GFP*; VLMi: ventral lateral midbrain exposed to current.

## Competing interests

The authors declare that they have no competing interests.

## Authors' contributions

EKF, EG, and DC devised experiments. EKF carried out electroporation and isolation of VLM from experimental embryos, analysis of microarray data and wrote the manuscript. EG carried out isolation of VLM from experimental embryos and advised on the manuscript. DC carried out RNA extraction, microarray experiments, microarray analysis and advised on the manuscript. ML provided financial support for the project. All authors read and approved the final manuscript.
